# Gastrointestinal angiodysplasia in three Saudi children

**DOI:** 10.4103/0256-4947.51786

**Published:** 2009

**Authors:** Ali Al-Mehaidib, Saleh Alnassar, Ali S. Alshamrani

**Affiliations:** a From the Department of Pediatrics, Asir Central Hospital, Abha, Saudi Arabia; b From the Department of Surgery, King Faisal Specialist Hospital & Research Centre, Riyadh, Saudi Arabia; c From the Department of Pediatrics, Asir Central Hospital, Abha, Saudi Arabia

## Abstract

Angiodysplasia is a term used to describe distinct gastrointestinal mucosal ectasias that are not associated with cutaneous lesions, systemic vascular disease or a familial syndrome. Seventy-seven percent of angiodysplasia are located in the cecum and/or ascending colon. Fifteen percent are located in the jejunum and/or ileum and the remainder are distributed throughout the alimentary tract. Most commonly, the angiodysplastic lesions are typically seen in elderly patients of both genders, although gastric and duodenal lesions have been reported occasionally in subjects within the third decade of life. However, data on infants and children are scarce. We describe three cases (ages 7 days, 2 years, and 5 years) who presented to our unit with gastrointestinal bleeding. One of these patients developed moderate-to-severe symptoms and was blood-transfusion dependent. She was misdiagnosed as having inflammatory bowel disease and underwent a total colectomy and ileoanal anastomosis. The other two patients were managed conservatively for up to 5 years with no further bleeding.

Angiodysplasia is the term used to describe distinct gastrointestinal mucosal vascular ectasias that are not associated with cutaneous lesions, systemic vascular disease or a familial syndrome. These lesions may be flat, red spots (2-5 mm) or slightly raised. The lesions have many similarities to those of the telangiectasias, but angiodysplasia is not a component of a systemic, metabolic or hereditary disease with other manifestations.^1^ Seventy-seven percent of angiodysplasias are located in the cecum and/or ascending colon. Fifteen percent are located in the jejunum and/or ileum and the remainder are distributed throughout the alimentary tract.^2^ The exact cause of angiodysplasias is unknown, but the widely accepted theory is that they are related to degenerative changes of the small blood vessels associated with aging. Other theories include longt-term local hypo-oxygenation of the microcirculation from cardiac, vascular or pulmonary disease.^1^ Mucosal hypoperfusion from cardiac disease was postulated to be the underlying cause for development of angiodysplasia, but echocardiogram studies indicated that only a few patients with angiodysplastic lesions had significant valvular heart disease such as aortic stenosis.^2^ In many instances angiodysplasia disappear following aortic valve replacement.^3^ Roskell et al demonstrated a relative deficiency of collagen type IV in mucosal vessels in angiodysplasia compared with controls.^4^ Angiodysplasia has been reported in adults, but data on infants and children are scarce. We describe three cases that presented to our pediatric gastroenterology unit.

## CASE 1

**Figure 1:**
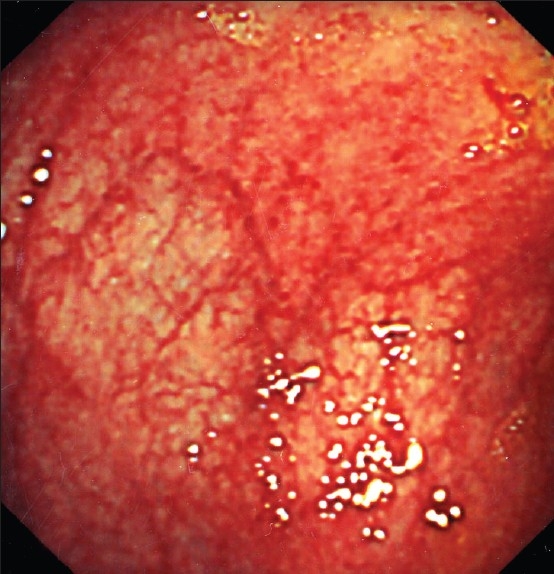
Diffuse bleeding form tiny angiodysplastic lesions over the sigmoid colon.

**Figure 2:**
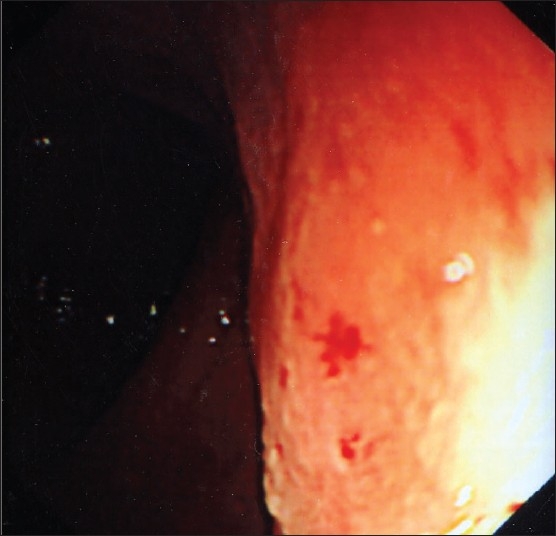
Typical fold processes of dilated superficial mucosal capillaries in the sigmoid colon.

**Figure 3:**
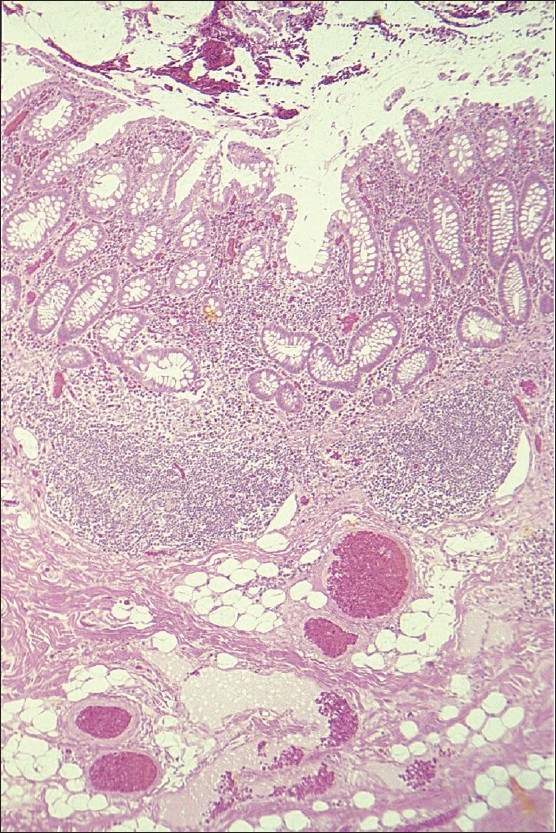
Colonic surgical specimen showing angiodysplastic lesions.

The first case was a 5-year-old Saudi female who had severe, frequent bloody diarrhea for two years associated with intermittent abdominal pain, poor appetite and failure to gain weight. At age of 3 years, she was treated at her local hospital for amoebic dysentery with antibiotics (sulfamethoxazole-trimethoprim and metronidazole) with no obvious improvement, followed by eliminating dietary milk products with some improvement, but she later had a recurrence of symptoms. Sigmoidoscopy in the local hospital showed a normal looking mucosa and biopsies showed chronic non-specific colitis; subsequently, the patient was started on prednisolone empirically with good response, but whenever the steroid dose was tapered, she started to bleed again from the rectum. She was referred to our center as having steroid-dependent colitis for further management. Examination revealed a pale, non-thriving child with both weight and height below the fifth percentile for age. The rest of the systemic examination was normal. Her hemoglobin was 5.3 mg/dL (normal, 11.0-14.0 mg/dL), mean corpuscular volume was 64.1 fL (normal, 80.0-94.0 fL), mean corpuscular hemoglobin was 19.9 pg/cell (normal, 27.0-32.0 pg/cell), the erythrocyte sedimentation rate was 31 mm/h, and stool for occult blood was positive, serum albumin was 38 g/L (normal, 32-46 g/L), and total protein was 69 g/L (normal 65-81 g/L). Colonoscopy revealed tiny angiodysplastic lesions in the rectum, sigmoid colon, and the ascending colon (Figure 1). The transverse colon and cecum again showed small hemorrhagic angiodysplastic lesions with typical fold processes of dilated superficial mucosal capillaries. No ulcers or erosions were seen (Figure 2). The upper gastrointestinal endoscopy was normal. The steroid was tapered and stopped. She became blood transfusion-dependent, requiring one transfusion every 3 to 4 weeks. With involvement of the whole colon with angiodysplastic lesions, a normal upper gastrointestinal endoscopy and with moderate-to-severe symptoms requiring multiple blood transfusion, the patient underwent total colectomy and ileoanal anastomosis with a smooth post-operative course. Histological examination was consistent with angiodysplastic lesions in different parts of the colon (Figure 3). Follow-up in the clinic up to 3 years post-surgery showed that the patient had no more rectal bleeding. Her growth improved and her hemoglobin stabilized at >10 mg/dL.

## CASE 2

A 2-year-old male was admitted at 5 months of age with fever and bronchopneumonia. He was treated with antibiotics and improved. Two weeks post-discharge, the infant developed hematemesis and melena. He was hemodynamically stable with a hemoglobin of 6.2 mg/dL (normal, 11.0-14.0 mg/dL) and needed blood transfusion. The liver and renal function tests were unremarkable. Upper gastrointestinal endoscopy in a local hospital revealed angiodysplasia at the greater curvature of the stomach with some oozing points. There were no esophageal varices and no ulcers. He was referred to our center with recurrent hematemesis for further management. At the age of 1 year, upper GI endoscopy at our center revealed a normal esophagus with few angiodysplastic lesions over the body of the stomach while the duodenum was normal. Colonoscopy showed dilated blood vessels consistent with angiodysplastic lesions from the rectum to the mid-ascending colon. He was treated conservatively, without surgical intervention. He had no further bleeding and was placed on iron therapy. At the age of 6 years, he was seen in the clinic and had a weight and height above the tenth percentile. His hemoglobin was 13.3 mg/dL while maintained on an iron supplement.

## CASE 3

A 7-day-old male infant, a product of a full-term in vitro fertilization pregnancy was delivered by lower segment cesarean delivery secondary to fetal distress by a mother with primary infertility for six years. The baby was dusky after birth and was resuscitated with oxygen using an Ambu bag, but did not need intubation to resolve transient tachypnea of the newborn. He was fed with artificial feeds (cow's milk formula). On day 3, he had a bloody stool, with no abdominal distension and audible bowel sounds. He did not appear septic and vital signs were normal. He was placed on nil per OS nothing by mouth status and started on intravenous ampicillin and gentamicin after appropriate cultures were taken. An abdominal x-ray was unremarkable. His complete blood count, including platelet count and coagulation profiles were normal. Stools on day 6, 7 and 8 were positive for occult blood. He underwent colonoscopy on day 8, which revealed angiodysplastic lesions oozing blood. The lesions increased in number as the scope advanced proximally to the sigmoid and descending colon and decreased toward the transverse and ascending colon. Bleeding appeared whenever air or water were applied directly over the lesions. The mother refused upper GI endoscopy for evaluation of the rest of the gut. The patient was treated with oral iron supplementation and discharged home. On follow-up, he had no more bleeding and received iron therapy as needed. At 3 years of follow-up, the baby was growing well with no rectal bleeding and had a stable hemoglobin of 11 mg/dL.

## DISCUSSION

Angiodysplasia is an increasingly recognized disorder in adults and the prevalence appears higher than was originally suspected.^5^ Among adults, angiodysplasia of the stomach or duodenum was found in 1% to 2% of 25 consecutive subjects undergoing upper gastrointestinal endoscopy for a variety of indications.^5^ Angiodysplasia is found during colonoscopy at slightly higher rate of 3% to 6% of subjects undergoing the procedure for a variety of indications.^6^,^7^ Angiodysplasia can be an incidental finding in nearly half of the cases in which it is detected.^6^ Most commonly, the angiodysplastic lesions are typically seen in elderly patients of both genders, although gastric and duodenal lesions occasionally have been reported in subjects during the third decade of life.^5^,^8^ Data are scarce in infants and children, and to our knowledge, these are the first case reports among Saudi children. As with upper tract lesions, very young subjects with colonic angiodysplasia have also been described.^9^ Angiodysplasia may present with maroon-colored stool, melena, and hematochasia as in Case 1 and 3 or hematenesis as in Case 2. In 10% to 15% of patients, iron deficiency anemia is observed and stools are intermittently positive for occult blood (as in Case 2 and 3).^6^ Fifteen percent of patients present with massive hemorrhage as in Case 1, and bleeding stops spontaneously in more than 90% of cases (as in Case 2 and 3).^7^ While there are many modalities for diagnosis of angiodysplasia such as selective mesenteric angiography, 99m Tclabeled red blood cells, 99m Tc-labeled sulfur colloid, CT angiography, air contrast enema and wireless capsule, colonoscopy is the most common method of diagnosis as well as therapy. The sensitivity of colonoscopy exceeds 80% when the lesions are located in the area examined by colonoscopy.^7^

In Case 1, a limited sigmoidoscopy was performed and she was misdiagnosed as having inflammatory bowel disease. Endoscopic forceps biopsy has revealed characteristic histopathological features of angiodysplasia in only 31% to 60% of specimens.^10^ Angiodysplasia is infrequently detected by visual inspection of the serosal side of the bowel wall.^11^ In 1 of 39 adult cases (2.6%), the diagnosis was made during surgical exploration, and 14 (35.8%) of these individuals underwent 21 non-diagnostic surgeries prior to their evaluation. A conservative approach to hemodynamically stable patients is recommended as bleeding ceases spontaneously in the majority of the cases (as in Case 2 and 3). Gastric and duodenal angiodysplastic lesions are managed with endoscopic obliteration techniques. In one study, the blood replacement requirements for a group of 13 patients decreased by 50% with 4 patients requiring no further transfusion.^12^ Endoscopic laser photocoagulation is a successful modality in controlling bleeding from colonic angiodysplasia. However, complications occur in as many as 15% and are more common when the Nd: Yag Laser (neodymium-doped yttrium aluminium garnet; Nd:Y3Al5O12) is used in the right colon.^10^ In a pilot study, 14 patients bleeding from angiodysplasia and 4 bleeding from Dieulafoy lesions located in the small bowel observed that endoscopic band ligation achieved hemostasis in a single session in all patients.^13^

Angiodysplasia that presents with acute hemorrhage can be effectively controlled with embolization, although it is seldom needed, as in our three cases. Selective infusion of vasopressin is less effective than embolization because of a higher bleeding rate. However, intra-arterial vasopressin can control massive lower gastrointestinal bleeding in 70% to 91% of patients, but bleeding recurs after discontinuation of vasopressin in 22% to 71% of patients.^10^ Octreotide treatment should be considered in patients with refractory gastrointestinal bleeding due to angiodysplasia, in particular in those who need anticoagulant treatment.^14^ Octerotide has shown a beneficial response in the long-term management of patients with gastrointestinal angiodysplasia.^15^,^16^

Colectomy for angiodysplasia is a second-line therapy after endoscopic ablation if endoscopic therapies are not applicable as was the case in our first case, and if life-threatening hemorrhage occurs. In one report, right hemicolectomy resulted in 63% of subjects remaining free of intestinal bleeding for a mean of 3.6 years, and 37% had some degree of recurrent bleeding.^12^ Re-bleeding after hemicolectomies occurs in 5% to 30%,^13^ which is much less than with endoscopy technique. In general, the prognosis is favorable because most angiodysplasias spontaneously cease bleeding in more than 90% of cases,^16^ as in Cases 2 and 3.

In summary, gastrointestinal angiodysplasia is a rare disorder seen in infants and children. In the majority of cases, bleeding stops spontaneously and no further treatment is required. A minority of cases present with moderate-to-severe symptoms needing frequent blood transfusions, and endoscopic and/or surgical intervention may be inevitable.
